# Potential Effects of Medicinal Plants and Secondary Metabolites on Acute Lung Injury

**DOI:** 10.1155/2013/576479

**Published:** 2013-10-09

**Authors:** Daniely Cornélio Favarin, Jhony Robison de Oliveira, Carlo Jose Freire de Oliveira, Alexandre de Paula Rogerio

**Affiliations:** ^1^Departamento de Clínica Médica, Laboratório de ImunoFarmacologia Experimental, Instituto de Ciências da Saúde, Universidade Federal do Triângulo Mineiro, Rua Manoel Carlos 162, 38025-380 Uberaba, MG, Brazil; ^2^Instituto de Ciências Biológicas e Naturais, Universidade Federal do Triângulo Mineiro (UFTM), Uberaba, MG, Brazil

## Abstract

Acute lung injury (ALI) is a life-threatening syndrome that causes high morbidity and mortality worldwide. ALI is characterized by increased permeability of the alveolar-capillary membrane, edema, uncontrolled neutrophils migration to the lung, and diffuse alveolar damage, leading to acute hypoxemic respiratory failure. Although corticosteroids remain the mainstay of ALI treatment, they cause significant side effects. Agents of natural origin, such as medicinal plants and their secondary metabolites, mainly those with very few side effects, could be excellent alternatives for ALI treatment. Several studies, including our own, have demonstrated that plant extracts and/or secondary metabolites isolated from them reduce most ALI phenotypes in experimental animal models, including neutrophil recruitment to the lung, the production of pro-inflammatory cytokines and chemokines, edema, and vascular permeability. In this review, we summarized these studies and described the anti-inflammatory activity of various plant extracts, such as *Ginkgo biloba* and *Punica granatum*, and such secondary metabolites as epigallocatechin-3-gallate and ellagic acid. In addition, we highlight the medical potential of these extracts and plant-derived compounds for treating of ALI.

## 1. Introduction 

Acute lung injury (ALI) and its severe form, acute respiratory distress syndrome (ARDS), were first described in 1967 by Ashbaugh et al. [1] in patients with acute onset of tachypnea and hypoxia and the loss of compliance after a variety of stimuli [2–5]. According to the American-European Consensus Conference (AECC) ARDS was recognized as the most severe form of acute lung injury (ALI), a form of diffuse alveolar injury. In addition, the Berlin definition modified the AECC definition and divided ALI into the independent categories of ALI non-ARDS and ARDS alone [6, 7]. ALI is a life-threatening syndrome that causes high morbidity and mortality [8–12]; however, the worldwide incidence is variable, reaching, for example, 64.2 to 78.9 cases/100,000 person-years in the United States and 17 cases/100,000 person-years in Northern Europe, with an estimated 74,500 deaths annually [13]. Patients admitted to intensive care units are most affected by ALI (1 in 10) [14]. However, individuals with multiple comorbidities, chronic alcohol abuse, or chronic lung disease also present a high risk of developing ALI [15, 16]. The causes of ALI may be direct, such as *pneumonia*, inhalation injury, aspiration of gastric contents, inhalation injury, chest trauma, and near drowning, or indirect, such as sepsis, burns, pancreatitis, fat embolism, hypovolemia, and blood transfusion [8, 14]. The pathogenesis of ALI involves increased permeability of the alveolar-capillary membrane, accumulation of protein-rich fluid in the airspaces, pulmonary edema, and pulmonary infiltration of neutrophils, mainly bilateral, resulting in poor lung compliance, diffuse alveolar damage, and, consequently, acute hypoxemic respiratory failure [8, 17–23]. 

The inflammatory process of ALI can be classified into three stages: exudative, proliferative, and fibrotic stages [4, 9, 14]. The exudative stage is characterized by intense neutrophilic infiltrate, edema, and protein-rich fluid due to pulmonary capillary leakage [14]. The proliferative stage ensues as a consequence, the development of which is marked by proliferation and phenotypic changes in type II alveolar cells and fibroblasts [20]. In the absence of recovery, the fibrotic stage develops, which is characterized by diffuse fibrosis and modulation of the structural architecture remodeling of the lung. These stages characterize the chronic phase of ALI, leading to the formation of fibrotic scarring in the lung [9, 14].

Although inflammation is essential for the maintenance of tissue homeostasis and protection against infections, uncontrolled inflammation may contribute to lung damage, a characteristic phenomenon of several inflammatory disorders, including ALI [24–26]. In ALI airway inflammation, neutrophils are the first cells to be recruited and are the predominant cause of tissue damage [25, 27, 28], and their persistence is associated with a poor ALI prognosis [27–29]. The increased accumulation of neutrophils is associated with the exacerbation/amplification of inflammation and, consequently, of lung lesions due to the release of a complex network of proinflammatory mediators, such as cytokines (interleukin (IL)-1*β*, tumor necrosis factor (TNF)-*α*, IL-6, and IL-8), chemokines chemokine (C-X-C motif) ligand (CXCL)-8, CXCL-1, CXCL-5, and chemokine (C-C motif) ligand (CCL)-2), proteases (elastases, collagenases, cathepsin G, and metalloproteinases), and oxidants (hydrogen peroxide and superoxide), and the accumulation of necrotic material [4, 24–26, 28–31]. Interestingly, an increase in such anti-inflammatory cytokines as IL-10 is also observed in ALI [32–34]. Thus, the balance of proinflammatory and anti-inflammatory mediators could coordinate the evolution or resolution of ALI. The resolution of inflammation is an active process and requires the activation of endogenous mechanisms, such as the biosynthesis of lipid mediators with proresolution activity, interaction between cells (hematopoietic and/or structural cells), and activation of cellular processes (e.g., apoptosis, phagocytosis) to maintain homeostasis [35–38]. Resolution includes the steps of (a) the inhibition of polymorphonuclear cell (neutrophil) infiltration, (b) return to normal vascular permeability, (c) clearance of polymorphonuclear cells (mainly by apoptosis), (d) infiltration of monocytes/alternatively activated macrophages, and (e) removal of apoptotic neutrophils, microorganisms, allergens, and foreign agents by macrophages [36, 38–42]. Clearly, resident and recruited macrophages play an important role in the clearance of injured tissues, debris, and apoptotic cells and are therefore important for the resolution of inflammation [36, 38]. Specifically, the resolution in ALI is characterized by the removal of neutrophils in the lung and the restoration of epithelial barrier function [38, 43]. Animal models have not been developed that fully to resemble human ALI but are quite useful for the better understanding of airway inflammation and the development of ALI [18, 44]. ALI experimental models in mouse, rat, rabbit, and guinea pigs are reported in the literature using different triggers, such as lipopolysaccharide (LPS), live bacteria, acid aspiration, and others. A more detailed description of the most commonly used ALI models and their characteristics can be found in Table 1.

The considerable progress made through the use of molecular and cellular assays together with knockout and transgenic animals has contributed significantly to the understanding of the genetic, tissue-specific, and immunological factors that contribute to the development of ALI [45–52]. Nevertheless, no therapeutic agents have demonstrated a clear benefit in ALI treatment [41], and corticosteroids have been used for treatment of ALI for many years [18, 53]. Besides, the disappointing results of a series of clinical trials treatment of ALI or patients at risk for ARDS using corticosteroids as well as the increase of the risk of infection and other adverse effects, the administration of corticosteroids might improve the injured tissue due to their anti-inflammatory effect [54]. Thus, the development of new compounds that exhibit similar therapeutic potential with reduced adverse effects is necessary for the continuous treatment of ALI. Furthermore, agents of natural origin that induce very few side effects should be considered for use as therapeutic substitutes or as complementary treatments to existing therapies. In addition, natural compounds may even form the basis of new drugs for the treatment of diseases [55–58]. In the course of a continued search for bioactive natural products derived from plants (secondary metabolites), several groups, including our own, have successfully employed experimental models to screen the pharmacologic activities of plant extracts and isolated compounds (secondary metabolites) [59–62]. Within this context, we and others have demonstrated that many plant extracts and secondary metabolites have the potential to be used in ALI treatment [8, 60, 63]. In a clinical trial in patients with severe pulmonary hypertension during extracorporeal circulation, Xu et al. [64] demonstrated that composite Rhodiolae (herbal plant) reduced the occurrence rate of acute lung injury and its mortality. In addition other studies were carried out with plant extracts (Table 2) and plant-derived substances (Table 3) in ALI experimental models. 

*Ginkgo biloba* L. (Ginkgoaceae) is one of the most well-known plants in Chinese culture and has been used for therapeutic purposes for approximately 1,000 years. Its extracts are marketed worldwide to prevent or delay cognitive impairment associated with aging or neurodegenerative disorders [62, 65, 66]. In addition, *G. biloba* leaves have been used for the treatment of airway diseases, such as asthma and bronchitis [67]. In a bleomycin-induced acute lung injury rat model, a *G. biloba* leaf extract (EGb 761) reduced the responsiveness and diminished the occurrence of further reduction in the vasoconstrictor response of the pulmonary artery due to 5-hydroxytryptamine (5-HT). Furthermore, EGb 761 normalized bleomycin-induced alterations in the measured lung tissue biochemical markers [68]. Additionally, in another study, EGb 761 reduced protein leakage, neutrophil infiltration, myeloperoxidase (MPO, a heme enzyme present in the primary granules of neutrophils), and metalloproteinase (MMP)-9 activities in an LPS-initiated ALI rat model. These effects were associated with an inhibition of the activation of the nuclear factor-kappa B (NF-*κ*B) pathway. In LPS-induced acute lung injury rat model, *G. biloba* extract reduced the recruitment of leukocytes to bronchoalveolar lavage fluid (BALF) and the pulmonary permeability. In addition, besides reducing other parameters, *G. biloba* extract also reduced the blood TNF-*α* concentration and MPO in lung tissues [69]. Therefore, *G. biloba* appears to have potential to be used in the treatment of inflammation in ALI. 

Another plant with antineutrophilic potential is *Lafoensia pacari* Jaumes St. Hilaire (Lythraceae), the extract of which is traditionally used by the population of Mato Grosso state, Brazil, to treat inflammation and gastric ulcers [70, 71]. In a clinical trial, however, *L. pacari* methanolic extract failed to eradicate *Helicobacter pylori* in dyspeptic urease-positive patients, even though the extract was well tolerated, and about 74% of patients had partial improvement of dyspnea, and 42% had full improvement of dyspnea in patients treated with extract of *L. pacari* [72]. Employing the asthma model induced by *T. canis *infection or the ovalbumin-induced asthma model, our group demonstrated that oral treatment with an ethanolic extract of *L. pacari* decreased the number of eosinophils and neutrophils recruited to BALF [73, 74]. In an attempt to identify the molecule(s) responsible for the antieosinophil and antineutrophil activity of the *L. pacari* extract, we used a mouse model of peritonitis induced by exposure to the F1 fraction of the *H. capsulatum *yeast wall [75]. This model of acute and localized eosinophilia and neutrophilia was suitable for the bioassay-guided fractionation of the *L. pacari *extract, and we were able to isolate and chemically characterize ellagic acid (a polyphenol) as the major active component in the extract [61]. We showed that *L. pacari* extract as ellagic acid was able to reduce the number of eosinophils and neutrophils in this model [62]. We recently demonstrated that ellagic acid displayed anti-inflammatory properties by decreasing the severity of HCl acid-initiated ALI, accelerating the resolution of inflammation, and decreasing the cyclooxygenase-2 (COX-2) inhibitor-induced exacerbation of inflammation [60]. Ellagic acid reduced several inflammatory parameters, including vascular permeability alterations and neutrophil recruitment to BALF and the lung. In addition, ellagic acid reduced the proinflammatory cytokine IL-6 and increased the anti-inflammatory cytokine IL-10 in BALF without downregulating the NF-*κ*B and activator protein 1 (AP-1) signaling pathways [60]. 

Pomegranate (*Punica granatum*) extracts, which have been used for centuries for medical purposes, contain also ellagic acid, and studies with pomegranate extract have demonstrated the anti-inflammatory effects in an experimental model of ALI (LPS-initiated) by reducing MPO in the lungs of mice [76]. Together, these findings suggest that ellagic acid has potential anti-inflammatory effects for the resolution of ALI inflammation.

Flavonoids are the best studied class of plant metabolites. Indeed, the search term “flavonoids” yielded more than 64,786 entries in the U.S. National Library of Medicine's Medline database accessed using PubMed in May 2013. Flavonoids occur naturally in fruits and vegetables, such as onions, apples, grapes, and nuts and are therefore commonly part of the human diet [77]. These compounds are also a component of disease treatment (phytotherapy), as they are present in the seeds, stems, barks, roots, and/or flowers of several medicinal plants [78]. Flavonoids have shown a wide range of therapeutic properties in clinical and preclinical studies, including, but not limited to, antioxidant, anticancer, antiinflammatory, and antiallergy activities [79–82]. Luteolin, a widely distributed flavonoid, has been reported to exhibit anti-inflammatory, antioxidant, and anticarcinogenic activities [83]. Luteolin was reported to reduce several hallmarks of ALI (LPS-initiated): leukocyte infiltration, histological changes, lung tissue edema, protein extravasation, MPO activity in lung tissue, TNF-*α*, keratinocyte-derived chemokine (KC), IL-6, and intercellular cell adhesion molecule-1 (ICAM-1) production, as well as inducible nitric oxide synthase (iNOS) and COX-2 expression in the lung [84]. Additionally, the expression of surface markers CD11b and Ly6G on neutrophils was reduced [85]. Luteolin also reduced N-Formylmethionyl-leucyl-phenylalanine (fMLP)-induced neutrophil chemotaxis and respiratory burst after LPS challenge and reduced LPS-induced activation of the NF-*κ*B pathway, possibly via mitogen-activated protein (MAP) kinase (MAPK) and serine/threonine-protein kinases (AKT) [86]. These findings suggest that luteolin has potential anti-inflammatory effects for ALI treatment.

Green tea, from *Camellia sinensis* L. (Theaceae), is widely consumed around the world and is prepared by drying and steaming fresh tea leaves. Flavonoids are the major secondary metabolites found in green tea, with epigallocatechin-3-gallate being the most abundant. In acute lung injury induced by oleic acid in mice, epigallocatechin-3-gallate reduced the lung index, blood TNF-*α* concentration, and the phosphorylation of p38 MAPK [87]. In another study using LPS-initiated ALI in mice, epigallocatechin-3-gallate demonstrated an anti-inflammatory effect by reducing neutrophil recruitment in the lung and the production of TNF-*α* and macrophage inflammatory protein (MIP)-2, most likely via reduced extracellular-signal-regulated kinase (ERK)1/2 and c-Jun N-terminal kinase (JNK) phosphorylation in the lungs [63]. Therefore, epigallocatechin-3-gallate might constitute an attractive molecule with potential interest for the treatment of ALI.

The discovery of curcumin, the principal pigment of turmeric, dates from approximately two centuries ago when Vogel and Pelletier isolated a pigment of “yellow coloring matter” from the rhizomes of *Curcuma longa* (turmeric) [88–91]. Curcumin is present in the human diet and has been consumed for medicinal purposes for thousands of years [92]. This polyphenol has been shown to possess activities in the animal models of many human diseases. Curcumin modulates various molecules, including transcription factors, adhesion molecules, cytokines, and chemokines [92]. Curcumin demonstrated a significant anti-inflammatory effect with a reduction of the main ALI phenotypes, which included the reduction of neutrophil recruitment and activation, lung edema, inflammatory, and cytokines, most likely via a reduction of the NF-*κ*B pathway in several ALI models. These models include sepsis-induced acute lung injury induced by cecal ligation and puncture surgery [93, 94], aspiration of polyethylene glycol and activated charcoal [95], intestinal ischemia/reperfusion (I/R) [96], bleomycin-induced lung injury [97], acute inflammation by *Klebsiella pneumonia* introduction [98], oleic acid-induced ALI [99], and LPS-induced acute lung injury [100]. These findings suggest that curcumin could be an interesting alternative for the ALI treatment.

The alkaloid theophylline is one of the oldest drugs in use in the management of obstructive airway diseases of diverse etiologies [101, 102], despite its weakness as a bronchodilator. However, the use of this alkaloid is often limited due to concerns regarding dose-related adverse effects, numerous drug interactions, and a narrow therapeutic index. In a chronic inflammatory lung injury model induced by LPS in guinea pigs, theophylline improved the airway injury and airway hyperreactivity induced by the repetitive exposure to LPS [103]. These findings suggest that theophylline has potential anti-inflammatory effects for the treatment of ALI inflammation.

In conclusion, ALI is a disease with high morbidity and mortality, and the current disease outcome has yet to be improved by pharmacologic treatment. Natural products and plant derivatives used in folk medicine are of vast medical importance due to their potential as a source of molecules with pharmacologic properties. Although active plant-derived secondary metabolites can be randomly discovered, the process is laborious, with a success rate on the order of 1 new product per 10,000 plants screened [62, 104]. In this review, we reviewed the effect of some plant extracts and their components on ALI experimental models. The important benefits obtained with curcumin, ellagic acid, and *Ginkgo biloba* extract reveal powerful effects in reducing most ALI phenotypes, including inflammatory infiltrate, vascular permeability, and edema. As outlined in this review, we propose that there are several extracts of plants and compounds isolated from them with anti-inflammatory effects in ALI. So, they demonstrate potential to be used in the preliminary testing in humans which can provide a new alternative for ALI therapy.

## Figures and Tables

**Table 1:** Animal models of lung injury.

Model	Characteristic inflammation	Animals	References
Acid aspiration	Rupture of the alveolar-capillary barrier with intense neutrophilic infiltrate [23, 56, 105, 106]	Mice Rats Rabbits	[42, 56, 107–110]
Bleomycin	Acute inflammatory injury, and reversible fibrosis [23, 111]	Mice Rats	[112–114] [20, 115]
Cecal ligation and puncture	Variable neutrophilic alveolar infiltrate and increased permeability [23, 43]	Mice Rats	[111, 116, 117] [118–120]
Hyperoxia	Epithelial injury and neutrophilic infiltration, followed by type II cell proliferation and scarring [23, 121–123]	Mice Rats	[124–127] [128]
Intrapulmonary bacteria	Increased neutrophilic alveolar infiltrate, interstitial edema, and permeability [23, 129]	Rabbits	[129]
Intravenous bacteria	Interstitial edema, neutrophils sequestration, and intravascular congestion [23, 130]	Mice	[131]
LPS	Neutrophilic inflammation with increased intrapulmonary cytokines [20, 23, 132]	Mice Rats Sheep	[20, 45, 59, 70, 132–135] [136]
Nonpulmonary ischemia/reperfusion	Increased microvascular permeability, neutrophils recruitment, edema, and sequestration in the lungs [23, 28]	Mice Rats	[28, 137–139]
Oleic acid	Neutrophilic inflammation, increased permeability, and edema [22, 23, 140]	Mice Rats	[141] [21, 22, 142]
Peritonitis by cecal ligation and puncture	Variable degrees Neutrophilic alveolar infiltrate and increased permeability [23, 143]	Rats Rabbits	[143, 144] [44]
Pulmonary ischemia/reperfusion	Increased pulmonary vascular permeability, neutrophil infiltration, and edema [23, 145]	Mice Rats Rabbits	[145–147] [148]

**Table 2:** Plants with anti-inflammatory effect on ALI.

Plant	Model of ALI	Doses	Relevant findings	Reference
*Bathysa cuspidata *	ALI in rats induced by Paraquat	200 and 400 mg/kg	↓ Lung edema	[149]
*Ginkgo biloba *	ALI in mice induced by LPS	10, 100, and 1000 mg/kg	↓ Leukocytes, PMN, MPO, and NF-*κ*B	[150]
*Panax notoginseng *	ALI in rats induced by intestinal ischemia/reperfusion	100 mg/kg	↓ Leukocytes, PMN, MPO, IL-8, and TNF-*α*	[151]
Sho-seiryu-to	ALI in guinea pigs induced by oleic acid	3 and 0.75 g/kg	↓ Leukocytes and total protein	[152]
*Viola yedoensis *	ALI in mice induced by LPS	2, 4, and 8 mg/kg	↓ Leukocytes, total protein, lung edema, and MPO	[153]

**Table 3:** Secondary compounds with anti-inflammatory effects.

Active compound	Class	Models of ALI	Doses	Relevant findings	Reference
Matrine	Alkaloid	ALI in mice induced by LPS	25, 50, and 100 mg/kg	↓ Lung edema, MPO, TNF-*α*, IL-6, and NF-*κ*B	[154]
Nicotine	Alkaloid	ALI in mice induced by LPS	0.2 and 0.4 mg/kg	↓ Lung edema, leukocytes, MPO, total protein, IL-1, IL-6, and TNF-*α*	[155, 156]
		ALI in mice induced by LPS	50, 250, and 500 mg/kg	↓ Lung edema, MPO, TNF-*α*, and IL-1*β*	[157]
Theophylline	Alkaloid	ALI in guinea pigs induced by LPS	3 and 30 mg/kg	↓ Leukocytes, and ↑ IL-10	[103]
Andrographolide	Diterpenoid	ALI in mice induced by LPS	1 and 10 mg/kg	↓ Lung edema, leukocytes, MPO, IL-6, TNF-*α*, IL-1*β*, and NF-*κ*B	[59]
Astragalin	Flavonoid	ALI in mice induced by LPS	50 and 75 mg/kg	↓ Leukocytes, neutrophils, IL-1*β*, TNF-*α*, and IL-6	[158]
Caffeic acid phenethyl ester	Flavonoid	ALI in rats induced by phosgene	50 mg/kg	↓ NF-*κ*B, and p38 MAPK	[159]
Cardamonin	Flavonoid	ALI in mice induced by LPS	10, 30, and 100 mg/kg	↓ Lung edema, TNF-*α*, IL-6, and p38 MAPK	[160]
Epigallocatechin-3-gallate	Flavonoid	ALI in rats induced by LPS	10, 50, and 100 mg/kg	↓ Lung edema, neutrophils, TNF-*α*, MIP-2, and ERK	[63]
	Flavonoid	ALI in rats induced by oleic acid		↓ Lung edema, TNF-*α*, and p38 MAPK	[87]
Kaempferol	Flavonoid	ALI in mice induced by LPS	100 mg/kg	↓ Leukocytes, neutrophils, MPO, TNF-*α*, IL-1*β*, IL-6, ERK, and NF-*κ*B	[161]
Luteolin	Flavonoid	ALI in mice induced by LPS	35 and 70 *μ*mol/kg	↓ neutrophils, MPO, MAPK, and NF-*κ*B	[85]
		ALI in mice induced by LPS	70, 35, and 18 *μ*mol /kg	↓ neutrophils, IL-6, TNF-*α*, and NF-*κ*B	[84]
Naringin	Flavonoid	ALI in mice induced by LPS	15, 30, and 60 mg/kg	↓ Leukocytes, neutrophils, MPO, TNF-*α*, and NF-*κ*B	[162]
		ALI in mice induced by Paraquat	60 and 120 mg/kg	↓ Leukocytes, TNF-*α*, TGF-*β*1, and MMP-9	[163]
Oroxylin A	Flavonoid	ALI in rats induced by LPS	15 mg/kg	↓ Neutrophils, TNF-*α*, and NF-*κ*B	[164]
Quercetin	Flavonoid	ALI in rats induced by LPS	1, 5, and 10 mg/kg	↓ MPO, TNF-*α*, and IL-8	[165]
Geniposide	Iridoide	ALI in mice induced by LPS	20, 40, and 80 mg/kg	↓ Leukocytes, MPO, IL-6, TNF-*α*, and ↑ IL-10	[166]
Genistein*	Isoflavonoid	ALI in rats induced by LPS	50 mg/kg	↓ Lung edema, PMN, MPO, and ICAM-1	[167]
		ALI in rats induced by LPS	50 mg/kg	↓ MMP-9, NO, and NF-*κ*B	[168]
Curcumin*	Polyphenol	ALI in rats induced by phosgene	50 and 200 mg/kg	↓ Leukocytes, Lung edema, total protein, MPO, TNF-*α*, and IL-8	[96]
		ALI in rats induced by sepsis	200 mg/kg	↓ TGF-*β*1	[94]
		ALI in rates induced by LPS		↓ Lung edema, neutrophils, and NF-*κ*B	[169]
		ALI in rats induced by intestinal ischemia/reperfusion	100 mg/kg	↓ Leukocytes	[96]
		ALI in rats induced by oleic acid	5, 10, and 20 mg/kg	↓ Lung edema, TNF-*α*, and ↑ IL-10	[99]
Ellagic acid	Polyphenol	ALI in mice induced by acid	10 mg/kg	↓ Lung edema, neutrophils, IL-6, COX-2, NF-*κ*B, and AP-1	[60]
Escin	Saponin	ALI in mice induced by LPS	0.9, 1.8, and 3.6 mg/kg	↓ MPO, TNF-*α*, and IL-1*β*	[170]
Glycyrrhizin	Triterpene	ALI in mice induced by LPS	10, 25, and 50 mg/kg	↓ Lung edema, neutrophils, MPO, COX-2, and iNOS	[171]
		ALI in mice induced by LPS	30, 10, and 3 mg/kg	↓ TNF-*α* and ↑ IL-10	[172]
Hydroxysafflor yellow A		ALI in mice induced by LPS	6, 15, and 37.5 mg/kg	↓ Leukocytes, lung edema, TNF-*α*, IL-1*β*, IL-6, p38 MAPK, and NF-*κ*B	[173]
		ALI in rats induced by oleic acid and LPS	20, 40, and 60 mg/kg	↓ Leukocytes, lung edema TNF-*α*, IL-6, IL-1*β*, and ICAM-1	[174]

*No access to the entire manuscript, no answer from the corresponding authors.

## Abbreviations

ALI: Acute lung injury
ARDS: Acute respiratory distress syndrome
BALF: Bronchoalveolar lavage fluids
CCL: Chemokine (C-C motif) ligand
COX-2: Cyclooxygenase-2
CXCL: Chemokine (C-X-C motif) ligand
EGb: *Ginkgo biloba* extract
HCl: Hydrochloric acid
IL: Interleukin
LPS: Lipopolysaccharide
MAPK: Mitogen-activated protein (MAP) kinase
MPO: Myeloperoxidase
NF-*κ*B: Nuclear factor-kappa B
TNF-*α*: Tumor necrosis factor-alpha
SOD: Superoxide dismutase
MDA: Malondialdehyde
TBX: Thromboxane
ROS: Reactive oxygen species
ERK: Kinase activated by extracellular signal
MIP: Macrophage inflammatory protein
MMP: Matrix metalloprotease
GR: Glucocorticoid receptor
iNOS: Inducible nitric oxide synthase
PMN: Polymorphonuclear.
